# Observations on Fever and Other Diseases of the South and West

**Published:** 1829-01

**Authors:** 

**Affiliations:** Tennessee


### Art. II.—*Observations on Fever and other Diseases of the South and West.* By Medicus.

### NO. I.

[The following communication which will be found to contain several animadversions on the opinions of two distinguished professors of Transylvania University, is from the pen of one of its own graduates—respectable in society and in the profession—who prefers to have it published anonymously. We see no reason for refusing his request.—Ed.]

I may be esteemed irreverent by some, because of my unwillingness to accept any of the present and popular opinions of disease, and to others I may seem arrogant, when offering to the public the views which I entertain. Were I here to express a total disregard for the opinion of all such as suppose me either irreverent or arrogant, I should at once defy the credulous and the majority of our profession. I should also act insincerely. These men—the credulous, in a great degree give the ebb and flow to popular opinion, and that does much in medicine, as well as in law, politics, and theology.

Popular opinion is the analytic crucible, if directed by science in its operation, which refines all theories and sanctions or condemns all courses and all customs. It has long since conceded to every man, the privilege of publishing his views, and science claims them at the hands of her votaries. As in Pharmacy no man has the exclusive right, in the eye of philanthropy, to prepare and vend any particular or useful composition, so in medicine, no man has the exclusive right to the quiet and undivulged enjoyment of any views connected with his profession.

All theories of the present day should be considered as common stock, the proceeds of which all are equally entitled to receive or reject. They are but flowers blooming on bygone hypothesies, and these but the scions of others, antecedent to themselves; which but for the loss of an occasional sprout, might be traced back to the days before the deluge. Time, however, has gathered such into her storehouse as are unnecessary for our improvement.

Having said this much about our mutual dependence and claims upon each other, and of the propriety of esteeming all theories and all facts as common stock, I hope not to be accused of plagiarism. Whatever the reader may discover herein, which he himself has seen elsewhere, I willingly confess is not my own. To it I set up no claims.

Before entering into the main design of this paper, I would remark that Health is the great object of our profession. That this depends upon, or is the result of the sound and normal action of all the divisions and subdivisions of every organ and tissue of the body. Life is the material agent which gives power and activity to those divisions and tissues,

A well balanced state of atmospheric air, is what affords the most adequate and the most necessary amount of this matter.* A properly regulated condition of the atmosphere, affording to each individual its requisite *quantum* of life or vitality, and this acting in sound organs and tissues, constitutes health, and is the natural condition of the body. This is the condition in which we were created originally, and which most usually obtains at our birth. The condition of health is the positive and natural state of the system. It is the active and motive condition of the body. Disease is the unnatural, inactive, immotive, and negative state. Health is natural. Disease the reverse or the opposite. Whatever is the reverse or opposite to nature, cannot really and positively exist. Disease then is not an actual condition of the body *in et per se.* It is parasitical, and depends upon a negative, or the absence of health for its prevalence; just as darkness prevails when light is extinguished, or as we become cold when heat is removed from us. As light may vary, from the splendid brilliancy of mid-day, to absolute darkness; as caloric may range in degree from the mountain melting heat of Enceladus' breath to the congelation of mercury, so health may prevail from the point of giving centrifugal and centripetal action to the proper tissues of the system, down to absolute and entire extinction. Were it not for the climatorial vicissitudes, whether dependent on different degrees of temperature or miasmatic pharmics I will not here enquire, and for the wear of all ponderable matter, health would be perpetual and old age universal. Accident would be the only outlet to life. Inasmuch as we can neither regulate the air nor give perpetuity to matter, it is proper next for us to enquire into the causes which produce a departure from health or a prevalence of disease. I do not intend so much to investigate the

* In some future hour I shall pen an essay designed to establish, first the truth of Life's being a material agent; second, that it is imponderable; third, that it is originally communicated to us by our parents and subsequently derived from the atmosphere; fourth, that it may and does exist in a latent as well as an active condition; and lastly, that it is the same in man, and in vegetables.

character of the impressive cause, as to ascertain the ubiquity of the primitive impression and its effects, as a cause of future symptoms and inconvenience.

Almost any of the popular works on the theory of fever affords satisfactory refutation of the former opinions and theories of disease; but in the Pathology and Therapeutics of Professor Cooke, of Transylvania, may be seen the most extensive exposition of old opinions that I have met with. Not choosing here to notice particularly many of those to which I have given reference, I shall advert to some composing the stock upon which the professor has engrafted his own theory. Hippocrates makes "disease to consist in an undue proportion of phlegm, blood, bile, and black bile." Sylvius says "that the cause of every continual fever is choler or lympha (under which I comprehend the juice of the pancreas, and spittle itself, inasmuch as they are carried to the heart) both vitious and raising an effervency in the right ventricle of the heart, whence the pulse is continually produced more frequent." Dr. Browne Langrish, in his Practice of Physic, says, "Obstructions are generally formed in the last series of the vessels, or in the ultimate branches of the sanguine and lymphatic arteries, and always arise from excess in the diameter of obstructing matter, above that of the canal through which it ought to flow." "When any of the juices stagnate in their respective vessels, or lie out of the verge of circulation, they will ferment and turn putrid from an intestine and fermentative motion, occasioned partly by the heat of the body, and partly by the heterogenity of their constitutive parts." "The blood in a state of health is a smooth demulcent liquor whose globules, both of the red and serous parts, are spherical and polished; but whenever the natural forms of the component parts are broken and destroyed, they must necessarily become angular, acrid, and pungent, and thereby more or less introduce an acrimonious quality which will *weaken* and destroy the vital powers."

"A disease," says Sydenham, "is no more than a vigorous effort of nature to throw off the morbific matter which arises either from such particles of the air as having a disagreement with the juices, insinuating themselves into the body, and mixing with the blood, taint the whole frame, or from different kinds of fermentations and putrefactions of humors detained too long in the body, for want of its being able to discharge and digest them on account of their too large bulk or unsuitable nature."

Our illustrious Rush says "From the facts and analogies which have been mentioned, I am led to conclude that the common forms of fever are occasioned simply by irregular action or convulsion in the blood vessels. This irregular action is of two kinds. The first is seated in the muscular coats of the blood vessels themselves. The second consists in an irregular distribution of blood to different parts of the body, particularly to the brain, lungs, liver, and spleen."

Professor Cooke makes cold and miasma to blacken the blood, to change the color of the skin and alvine evacuations, and the remote cause of fever to weaken the action of the heart.

The opinion of Sylvius that fever depends upon a vitious condition of the lymph and choler; of Langrish that the contents of the sanguine and lymphatic vessels, when they stagnate will become putrid, and, necessarily, angular, acrid, and pungent, producing an acrimonious quality which will *weaken* and destroy the vital powers; of Sydenham that disagreeing particles of the air mix with the juices and taint the blood; of Rush that fevers are occasioned by an irregular action or convulsion of the vessels; and of Cooke that the remote causes of fever, cold and miasma, produce dark blood, yellow skin, and green stools, are all kindred branches of the same family and are all wrong. They make disease to consist in one of the effects of itself. The blood and the juices are with them, all wofully disordered; but how they come so, or by what process is produced the yellow skin, green stools, black blood, or what causes an undue proportion of phlegm, blood, bile, and black bile, we are left to conjecture. On this subject Dr. Cooke of Transylvania, is as mute as Hippocrates of Cos. But more of "Cooke on the theory of fever," presently.

Professor Caldwell's opinion of fever is a corruption of Sydenham's. But read him. "The elements of fever afford incontestible evidence of the recuperative powers of the human system, and shew fevers to be nothing but an effort of those powers to restore health." *Elements of Fever*, sec. II.

"From whatever cause it may arise, or whatever course it may pursue in becoming so, fever when formed is a disease of the whole system. It invades the head, the trunk, and the extremities, deranging there the nerves, the muscles, the skin, the chylopoietic organs, and the glandular system generally. It injures also the brain and deranges of course the intellectual functions." *Ibid*, sec. III.

"Fever is nothing but an effort of nature to restore health. Fever is a disease of the whole system." Disease is sickness. Sickness of the whole system is here turned into a physician, and is making efforts to restore health, or to destroy itself. In these restorative efforts it will invade the head, the trunk, and the extremities, and derange the nerves, the muscles, the skin, and all the highly vitalized tissues of the system. It must injure the brain too, or its ultimate object,—to restore health,—cannot be affected. Then as fever is a physician and only at times needs a little adscititious aid, our duty plainly is to help it to invade the whole system, to derange the nerves and chylopoietic organs, and to injure the brain. This is confessedly no difficult task.

Reconcile these paragraphs to science, and it only is necessary, to the restoration of health that we should assist the elements of fever, first to invade the whole system; secondly to derange the nerves, the heart, and the absorbents. This amounts to active convalescence. The last indication in this course is just to injure the brain a little and suspend further attendance. But I must return to the "Theory of Fever," by Dr. Cooke.

"The remote cause of fever (miasma) does not produce its first effects upon the stomach. It affects (first) the color of the blood of every person exposed to its operation, and with this the color of the skin and passages." "Black blood does not stimulate the left ventricle of the heart like scarlet blood."

Blackness of the blood and the consequent weakness of the heart is the effect of miasma.

It would at least have been satisfactory, for the doctor to have said by what means or what process the blood, the skin, and the passages were changed in their color. Is it his opinion that the putrefactive process of vegetation yields to the air a quantity of gallic acid, and that this acid "insinuates itself into the juices which are stagnated and without the sphere of active circulation," unites with the iron of the blood and thus forms ink or produces the dark colour. I regret that he did not explain the manner of its production.

"Black blood does not stimulate the left ventricle of the heart like scarlet blood, and therefore the heart is weakened." "The remote causes of fever, cold and miasma weaken the action of the heart." "The remote cause of fever will blacken the blood." Why this confusion. Here the black blood is made to weaken the action of the heart; here also the remote cause of fever is made to weaken the action of the heart; and further the remote cause of fever will blacken the blood. In the proposition we see two effects, a weakened heart, and black blood from one (the remote) cause, and in them we see two causes, black blood and miasma, produce one effect, weakened action of the heart. If capable "*cognoscere causas rerum*," why this confusion? That black blood does not stimulate the left ventricle of the heart like scarlet blood, is proved by the experiments of Goodwin, "who attended carefully to the changes in the color of the blood, and in the corresponding contractions in the left auricle and ventricle of the heart. In all the examples, he observed, that when the blood which passed into the left auricle was florid, the auricle and ventricle contracted strongly, and the circulation went on as in health, but when the blood began to put on a shade of brown the contractions were diminished, and when it was black the contractions ceased."

The result of these experiments is what might have been predicted. The slow and diminished action of the heart are not the effect of black or brown blood, but they were produced, or both occurred from the injury done to the animal. As the heart became feeble in consequence of the shock sustained by the nervous system, the injury being so great as not to admit of a reactive effort, the heart was rendered, in common with the whole body, quite feeble, unable to propel the blood to the capillaries, and they also, injured in their powers, were ineffectual in their endeavours to perform on it their own peculiar office, or to send it to the lungs through the veins more effectually to be aerated or vitalized, hence its blackness. As the blood became brown or dark, the powers of nature were sinking; and as it was made black the heart ceased to contract; not because the blood was black, but because the animal was dead. All experiments which are in their effects so absolute, as to prevent reaction, produce death, and which give but a short time between the experiment and death, I imagine will give rise, as an effect antecedent to the final end, to the blackness of blood. In what manner, more effectually, this is brought about will be explained hereafter.

"Black blood weakens the action of the heart. If an accumulation of blood in the vena cava, amounts to that degree which gives rise to a convulsive agitation of the body, as in ague, whereby all the veins are compressed, and the blood urged vehemently to the heart, that organ excited by an increased quantity of its natural stimulus to increased action, fills the arteries, &c."

Accumulations in the vena cava are always of black blood; black blood weakens the hearts action; black blood is the natural stimulus to the heart, and will, when in increased quantity, excite the heart to an increased action. Now be it remembered, that the Professor in writing of black blood, always means that blood which is changed from a healthy, to an unnatural color, by the remote cause of fever. If a small amount of black blood will have an enfeebling effect upon the heart's action, what will an increased quantity of the same stimulus do? Assuredly an immediate suspension will be effected in its action. If the superior extremities of a laborer be fastened with a twine, it in some degree interferes with the use and power of exercising them, but bind him with a strong cord hand and foot, and his manipulating capacity is wonderfully increased! If the idea conveyed by the Professor is not tantamount to this, I egregiously misunderstand him; and if it be not heterodox, I misunderstand the subject. It is not entirely clear to me either, how black blood in the vena cava should be the natural stimulus to the heart. If the natural stimulus, why bring about an enfeebled action, or why produce an excited or increased action? It is represented as producing both. Neither weakened nor increased action is the natural action, and if the stimulus be natural, and the organ natural, why an action contrary to nature?

"The heart after long and violent exercise is disposed to relax its efforts, the accumulation of black blood, continually increasing whilst the heart is resting, gets, during rest, renewed vigor, and the necessary supply of stimulus having come to hand, it forthwith begins to increase its action:" notwithstanding the accumulated and black blood as well as the remote cause are both present, and both have a direct tendency to weaken its action.

It is no difficult matter to believe, that a few glasses of good Port wine will increase the action of the heart, and that as many pints will produce death; but is it so, that a debilitating power will, when applied in an increased volume, produce any other effect but that of greater weakness?

The doctor in his essay on fever apprehends no danger in the stage of excitement. That is all well enough. In the apyrexia, however, the system is in peril. Then it is, that congestions take place; that the stagnant juices take on an intestine and fermentative motion; become putrid, and turn black. And yet in his papers on autumnal fevers, his curative measures are all directed to the stage of excitement. He wishes them to operate in the active stage of fever. His evacuants, one drachm, half ounce, and ounce doses of calomel, or a tablespoonful of cathartic pills, must be given at such a time, as will cause the blac[illegible] yellow consistent excreta to pass in the act[illegible]. When he bleeds at all, it is done in the [illegible]. He deprecates the idea of blisters [illegible]s sinapisms, scorns tonics, and re[illegible] he can do any thing in the stage of excitement he does nothing in the stage of depression. It would, according to the view which I entertain, seem more scientific, if but one stage admits of danger, to direct our attention to the condition liable to produce or bring on injury, so that the system may be sustained and mischief avoided. If all the peril, is in the cold stage or remission; if the increased action of the heart is what relieves the engorgement, the fatal accumulation in the vena cava; why permit the cold stage to occur? why not keep the heart and arteries actively and constantly engaged, by the administration of some known and powerful stimulus, and thus prevent an accumulation, and at once cure the patient?

The Professor, in his willingness to exterminate all sympathetic communication or associated action, seems to have forgotten that the human body was possessed of any thing but a heart, arteries, venous cavities, black blood, and consistent passages: with him there is no secreting organ but the liver—no secretion vitiated but the secretion of bile. The lungs, the skin, and the kidneys are consigned, all together, to inutility. He regards neither the one nor the other of these important organs. From the contempt with which he treats the nervous tissue, he might be presumed as ignorant of it, as he is actually unmindful. The skin and the lungs being organs of such magnitude, both as regards their use and diffusion, and the nervous tissue being co-extensive with them in their amplitude, and these being the parts directly and constantly exposed to the operative power of the *vis noceus*, it seems a little strange, that the heart, the blood, and the liver, should be the camps first besieged by our enemy. And unless informed how the miasm or cold, can disorder these interior parts, without first injuring those through which it passes, I fear I shall continue to doubt the fact. Until convinced that the remote cause is endowed with a *tactus eruditus* or an elective power, so incredulo[illegible]ture, that I must remain sceptical of its capac[illegible] the inward parts of the system.

Acc[illegible]hich I have entertained for five or six y[illegible] which I have pursued the active [illegible] skin and the lungs possess the power to aerate or vitalize the blood. This they are enabled to do, by the assistance or presence of a sound and well balanced nervous tissue. The carbonic acid gas of the lungs and the perspirable matter of the skin, are equally secreted from the blood, by their respective organs, and together with the urine, they are as equally excrementitious, for they are deleterious when retained. In health the skin, the lungs, and the kidneys perform more excrementitious labor, than all the rest of the system. The suspension of their labor may be affected by the application of marsh poison, (if there be such a thing,) by cold, by heat, or by any thing which has the power of injuring the nervous system. Inasmuch as this is the system used by the body to give signs of impressive action, both to the individual impressed and to the physician; as the various secretions of the body depend for their proper and correct production upon a well and sound condition of their respective organs; as each organ is composed of a variety of tissues; as the nervous and vascular tissues pervade every organ; as the skin and lungs are always exposed first to the influence of noxious agents; as all active general disorders commence with a sense of coldness; as coldness is one of the invariable concomitants of the class of diseases called neuroses, I infer that the primary diseased action occurs in the nerves.

In another number of the Western Journal, I shall say what nerves are first disordered; what causes the cold fit of disease; account for the blackness of blood, and consider its claims to be regarded as a cause of reaction. This will prepare the way for the explanation of the pathology of the prevalent diseases of the South and West, and the correct mode of treatment.

*Tennessee, December*, 1828.

